# The risk of nontuberculous mycobacterial infection in patients with Sjögren’s syndrome: a nationwide, population-based cohort study

**DOI:** 10.1186/s12879-017-2930-7

**Published:** 2017-12-28

**Authors:** Wen-Cheng Chao, Ching-Heng Lin, Tsai-Ling Liao, Yi-Ming Chen, Chiann-Yi Hsu, Jun-Peng Chen, Der-Yuan Chen, Hsin-Hua Chen

**Affiliations:** 1 Department of Medical Research, Taichung Veterans General Hospital, 1650 Taiwan Boulevard, Sect. 4, Taichung, 40705 Taiwan; 2 Division of Chest Medicine, Department of Internal Medicine, Taichung Veterans General Hospital, Taichung, Taiwan; 3 Department of Business Administration, National Changhua University of Education, Changhua, Taiwan; 4 Institute of Biomedical Science and Rong-Hsing Research Center for Translational Medicine, Chung-Hsing University, Taichung, Taiwan; 5 Division of Allergy, Immunology, and Rheumatology, Department of Internal Medicine, Taichung Veterans General Hospital, Taichung, Taiwan; 6 School of Medicine, National Yang-Ming University, Taipei, Taiwan; 7 School of Medicine, Chung-Shan Medical University, Taichung, Taiwan; 8 Department of Medical Education, Taichung Veterans General Hospital, Taichung, Taiwan; 9 Institute of Public Health and Community Medicine Research Center, National Yang-Ming University, Taipei, Taiwan

**Keywords:** Sjögren’s syndrome, Nontuberculous mycobacteria, Immunosuppressant

## Abstract

**Background:**

Nontuberculous mycobacterial (NTM) infection in immunocompromized patients is currently a growing health concern, and we aimed to examine the relative risk of NTM infection in patients with Sjögren’s syndrome (SS) compared with that in non-SS individuals.

**Methods:**

We used the 2003–2012 Taiwanese National Health Insurance Research Database to identify 6554 incident SS cases during 2007–2012 and selected 98,310 non-SS controls matched (1:15) for age, gender, and the year of first SS diagnosis date after excluding those who had rheumatoid arthritis or systemic lupus erythematosus.

**Results:**

We identified four NTM-infected patients in the SS group (three in the first year) and nine in the non-SS group (three in the first year). SS patients had a higher incidence rate of NTM infection than that in non-SS individuals (IRR, 7.56; 95% CI, 2.33–24.55), especially during the first year (IRR, 16.05; 95% CI, 3.24–79.51). After adjusting for potential confounders, the risk of NTM infection was not increased in SS patients during the entire follow-up period or during the first year, but the risk increased in SS patients treated with immunosuppressants during the entire follow-up period (HR, 17.77; 95% CI, 4.53–69.61), especially during the first year (HR, 33.33; 95% CI, 4.37–254.23).

**Conclusion:**

An increased risk of NTM infection was found in SS patients treated with immunosuppressants during the first year after SS diagnosis.

**Electronic supplementary material:**

The online version of this article (10.1186/s12879-017-2930-7) contains supplementary material, which is available to authorized users.

## Background

Sjögren’s syndrome (SS), affecting approximately 1% of the general population, is a systemic chronic inflammatory disorder characterized by lymphocytic infiltrates in the exocrine glands and is primarily manifested with an insidious onset of dry eyes and dry mouth [1, 2]. Management of SS primarily consists of symptomatic treatment of sicca complaints and corticosteroids, while immunosuppressive agents, including azathioprine, methotrexate, and cyclophosphamide, are indicated for those with severe extraglandular systemic manifestations [3, 4]. Nontuberculous mycobacterial (NTM) infection is currently a growing health concern due to the globally increasing incidence and the need for prolonged therapy [5, 6]. Moreover, NTM is an opportunistic pathogen in immunocompromised patients, and the use of immunosuppressive agents in patients with rheumatic diseases may lead to an increased risk of mycobacterial infections [7–10]. A high risk of tuberculosis (TB) infection was reported in subjects with SS [11]; however, the risk of NTM infection in patients with SS has not been determined yet. We thus aimed to address the risk of NTM infection in patients with incident SS using a nationwide, population-based cohort.

## Methods

### Ethical statements

This study was approved by the Institutional Review Board of Taichung Veterans General Hospital, Taiwan (IRB number: CE14149B-2). All the personal data obtained were anonymized before analysis, and informed consent was thus waived.

### Study design and data source

In Taiwan, a single-payer National Health Insurance (NHI) program was launched on March 1, 1995. As of 2015, up to 99.6% of Taiwan’s population were enrolled in the NHI program [12]. The National Health Insurance Research Database (NHIRD) is the database of the program containing the registration files and original claims data for reimbursement. The Bureau of NHI (BNHI) was responsible for the management of NHIRD and released the data for research purpose. In this study, the ambulatory, inpatient, and enrollment data from the 2003–2012 NHIRD were used to identify patients with newly diagnosed SS. Furthermore, in Taiwan, patients with certain major illnesses, including cancer and certain autoimmune diseases, including SS, are issued a certificate of “catastrophic illness” and are thus exempt from copayment. In Taiwan, the diagnosis of SS is based on the classification criteria for SS proposed by the American–European Consensus Group in 2002 [13]. Patients with SS are issued the certificate of catastrophic illness for SS if two qualified rheumatologists validate their SS diagnosis after a review of patients’ medical records, laboratory data, and images. The NHIRD also has a catastrophic illness enrollment file for patients with catastrophic illness certificates, namely, the Registry for Catastrophic Illness Patient Database (RCIPD). In the present study, SS patients were enrolled only when their details were found in the RCIPD. Moreover, the NHIRD constructed a representative database of 1,000,000 individuals through random selection from all enrollees who received services in 2000 (Longitudinal Health Insurance Database, LHID2000). In the present study, the data of the non-SS control group were extracted through matching SS cases for age, gender, and the year of first SS diagnosis date from the LHID2000 database.

### Definitions of NTM infection

NTM infection was identified by the following International Classification of Diseases, 9th Revision, Clinical Modification (ICD-9-CM) codes for NTM: 031.0, 031.1, 031.2, 031.8, and 031.9 with a concurrent prescription of at least two anti-NTM drugs within 12 months of the diagnosis. Anti-NTM drugs consisted of amikacin, cefoxitin, ciprofloxacin, clarithromycin, doxycycline, ethambutol, imipenem, levofloxacin, meropenem, minocycline, moxifloxacin, rifabutin, rifampin, tigecycline, and streptomycin [14].

### Study samples

#### Incident SS patients identified from whole Taiwanese population

In this study, SS patients were defined as having at least three ambulatory visits or one hospital admission with a diagnosis of SS (ICD-9-CM code 710.2) and a catastrophic illness certificate for SS. From these SS patients, we excluded those who had SS diagnosis before 2007 based on the data between 2003 and 2006. To avoid the inclusion of patients with secondary SS resulting from rheumatoid arthritis (RA) or systemic lupus erythematosus (SLE), we excluded individuals who ever had a diagnosis of RA (ICD-9-CM code 714.0) or SLE (ICD-9-CM code 710.0) before the index date; therefore, the enrolled SS cases were newly diagnosed SS cases. The first date of visits with an SS diagnosis was selected as the index date. The index year was the year of the index date. We also excluded those who ever had ambulatory or inpatient visits with a diagnosis of NTM (ICD-9-CM codes 031.0, 031.1, 031.2, 031.8, and 031.9) before the index date.

#### Matched non-SS individuals selected from representative one million populations

From the LHID2000, we randomly selected non-SS individuals, matching SS cases (1:15) for age, gender, and the index year after exclusion of individuals who ever had ICD-9 codes for NTM or diseases of connective tissue (ICD-9-CM codes 710.x) during 2003–2012. The index date used for non-SS controls was the day of first ambulatory visit for any reason in the index year.

#### Potential confounders

Potential confounders used for adjustment in the Cox proportional regression model included age (<50 years, ≥50 years), gender, Charlson comorbidity index (CCI) (0, ≥1), and medications, including corticosteroids and immunosuppressants. The presence of comorbidity was defined as having at least three ambulatory visits or one inpatient visit with a corresponding ICD-9CM code within 1 year before the index date. The CCI, as adapted by Deyo et al. [15], was utilized to assess the level of general comorbid medical conditions. In this study, we also adjusted for medications that may increase infection risk, including corticosteroids and immunosuppressive agents, including azathioprine, methotrexate, and cyclophosphamide [16].

### Statistical analysis

Data were presented as mean ± standard deviation (SD) for continuous variables and as number (percentages) for categorical variables. The differences were analyzed using Student’s *t*-test for continuous variables and Pearson’s χ^2^ test for categorical variables. The primary dependent variable in this study was NTM incidence, and the incidence rates (per 100,000 person-year) and incidence rate ratios (IRRs) were analyzed. Kaplan–Meier method was used to compare the cumulative incidence of NTM between the SS group and the comparison group. A Cox proportional hazard model was conducted to estimate the hazard ratio (HR) of NTM infection in SS patients compared to that in non-SS individuals after adjustment for age, gender, CCI, and concomitant usage of corticosteroids in patients with SS. All the data were analyzed using statistical software version 9.3 (SAS Institute, Inc., Cary, NC, USA). A *P* value <0.05 was considered as statistically significant.

## Results

### Characteristics of the study population

A total of 6554 SS patients and 98,310 matched non-SS individuals were assessed (see Additional file 1: dataset for details). We found that SS patients had a higher CCI (0.5 ± 0.9 vs. 0.4 ± 1.0, *P* < 0.001) and were more likely to receive corticosteroids (69.6% vs. 39.0%, P < 0.001), cyclophosphamide (3.2% vs. 0.4%, P < 0.001), methotrexate (8.6% vs. 0.4%, P < 0.001), and azathioprine (12.5% vs. 0.1%, P < 0.001) (Table 1).

**Table 1:** Demographic data and clinical characteristics among patients

	Non- SS	SS	
	(*n* = 98,310)	(*n* = 6554)	*P*-value
Age, years (mean ± SD)	54 ± 14	54 ± 14	1
< 50 years	34,140 (34.7)	2276 (34.7)	
≥ 50 years	64,170 (65.3)	4278 (65.3)	
Gender			1
Female	86,760 (88.3)	5784 (88.3)	
Male	11,550 (11.8)	770 (11.8)	
CCI (mean ± SD)	0.4 ± 1.0	0.5 ± 0.9	<0.001
CCI group			
0	76,836 (78.2)	4541 (69.3)	
≥ 1	21,474 (21.8)	2013 (30.7)	
Medications during all follow-up period			
Immunosuppressants	837(0.9)	1349(20.6)	<0.001
Methotrexate	391(0.4)	563(8.6)	<0.001
Azathioprine	84(0.1)	819(12.5)	<0.001
Cyclophosphamide	425(0.4)	208(3.2)	<0.001
Steroid	38,348(39.0)	4559(69.6)	<0.001
Medications during the first year			
Immunosuppressants	413 (0.4)	955 (14.6)	<0.001
Methotrexate	214 (0.2)	360 (5.5)	<0.001
Azathioprine	41 (0.0)	572 (8.7)	<0.001
Cyclophosphamide	178 (0.2)	150 (2.3)	<0.001
Steroid	19,828 (20.2)	3588 (54.8)	<0.001

*Abbreviations*: *SS* Sjögren’s Syndrome

### Comparison of the incidence of NTM infection among SS patients with that among non-SS individuals

**Fig. 1:**
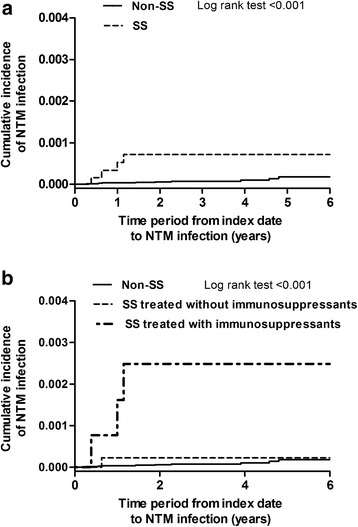
Kaplan-Meier survival curve for incidental NTM infection. **a** Categorized by SS and non-SS. **b** Categorized by non-SS, SS treated without immunosuppressants, and SS treated with immunosuppressants. NTM, nontuberculous mycobacteria; SS: Sjögren’s syndrome

Table 2 shows a comparison of the incidence rate of NTM infection among SS patients with that among non-SS individuals. A total of four SS patients (0.06%) developed NTM infection during the entire observation period. In the non-SS group, nine individuals (0.01%) developed NTM infection. The incidence rate of NTM infection was higher in the SS group (22 per 100,000 person-year) than that in the non-SS group (3 per 100,000 person-year), with an IRR of 7.56 (95% confidence interval [CI], 2.33–24.55). Given that the use of immunosuppressants, including methotrexate, cyclophosphamide, and azathioprine, might increase the risk of NTM infection, we further divided the patients with SS into those treated with and without immunosuppressants. We found that the incidence rate of NTM infection was significantly higher in SS patients treated with immunosuppressants (IRR, 24.98; 95% CI, 6.76–92.27) but not in SS patients treated without immunosuppressants (IRR, 2.45; 95% CI, 0.31–19.30), in comparison with non-SS individuals. We further investigated the correlation between SS and NTM infection using Kaplan–Meier estimates and found a marked increased incidence of NTM infection among SS patients (Fig. 1a), especially in those treated with immunosuppressants (Fig. 1b).

**Table 2:** Incident NTM infection categorized by the year after the diagnosis of SS

Variable	Total	Event (%)	Total person-years	Incidence Rate (/10^5^ years)	IRR (95%CI)
The entire period					
Non- SS	98,310	9 (0.01)	312,435	3	1
SS	6554	4 (0.06)	18,365	22	7.56 (2.33–24.55)
SS treated without immunosuppressants	5205	1 (0.02)	14,196	7	2.45 (0.31–19.30)
SS treated with immunosuppressants	1349	3 (0.22)	4169	72	24.98 (6.76–92.27)
Total	104,864	13 (0.01)	330,800	4	
1st year					
Non- SS	98,310	3 (0.00)	95,803	3	1
SS	6554	3 (0.05)	5970	50	16.05 (3.24–79.51)
SS treated without immunosuppressants	5599	1 (0.02)	5089	20	6.27 (0.65–60.33)
SS treated with immunosuppressants	955	2 (0.21)	881	227	72.51 (12.12–433.98)
Total	104,864	6 (0.01)	101,773	6	

Immunosuppressants: methotrexate, cyclophosphamide, or azathioprine. *Abbreviations*: *SS* Sjögren’s Syndrome, *CCI* Charlson comorbidity index

### Comparison of the incidence of NTM infection among SS patients with that among non-SS individuals during the first year of follow-up

Of the four NTM-infected SS patients, three (75.0%) developed NTM infection within the first year after SS diagnosis. Three (33.3%) of the nine NTM-infected non-SS individuals developed NTM infection within 1 year after the index date. Given that most of the NTM infections occurred in the first year among patients with SS, we calculated the IRR of NTM infection in SS patients compared to that in non-SS individuals within the first year after the index date. As shown in Table 2, a high IRR (16.05; 95% CI, 3.24–79.51) of NTM infection was observed in SS patients compared to that in non-SS individuals. Similarly, compared with non-SS individuals, the incidence rate of NTM infection was higher in SS patients treated with immunosuppressants (IRR, 72.51; 95% CI, 12.12–433.98) as well as in SS patients treated without immunosuppressants (IRR, 6.27; 95% CI, 6.27–60.33) (Table 2). In Kaplan–Meier estimates, we observed the highest risk of NTM infection in SS patients treated with immunosuppressants (Additional file 2: Figure S1A and B). Taken together, these data indicated that subjects with newly diagnosed SS, particularly those receiving immunosuppressants, had a higher incidence of NTM infection than that of non-SS individuals.

### Risk of NTM infection in patients with SS and in subgroups based on age, gender, and comorbidities

We next estimated the risk of NTM infection in SS patients using univariate and multivariable Cox proportional regression analyses. As shown in Table 3, during the entire follow-up period, a significantly higher risk of NTM infection was observed in SS patients receiving immunosuppressants compared to that in non-SS individuals (HR, 17.77; 95% CI, 4.53–69.61), but not in all SS patients (HR, 3.17; 95% CI, 0.70–14.24). As shown in Table 4, during the first year of follow-up, the risk of NTM infection was higher in SS patients than that in non-SS controls in the univariate analysis (HR, 16.06; 95% CI, 3.24–79.60). However, after adjusting for potential confounders, including age, gender, CCI, and usage of corticosteroids, we found that the association between SS and the risk of NTM infection did not reach statistical significance (HR, 5.22; 95% CI, 0.71–38.64) (Table 4, model A). SS patients treated with immunosuppressants during the first year of follow-up had an increased 1-year NTM infection risk (HR, 33.33; 95% CI, 4.37–254.23) (Table 4, model B) (See Additional file 3: Table S1 for data of all study period). Collectively, these data demonstrated a significantly increased 1-year risk of NTM infection in SS patients treated with immunosuppressive agents but not in those treated without immunosuppressants.

**Table 3:** Crude and adjusted hazard ratios for the association between variable and the risk of NTM infection during all follow-up period

	Univariate analysis		Model A		Model B	
	HR	(95%CI)	HR	(95%CI)	HR	(95%CI)
Group						
Non- SS	Reference	Reference	Reference	Reference	Reference	Reference
SS	7.66	(2.36–24.94)	3.17	(0.70–14.24)		
SS treated without immunosuppressants	2.47	(0.31–19.53)			1.95	(0.24–15.68)
SS treated with immunosuppressants	25.45	(6.88–94.05)			17.77	(4.53–69.61)
Age						
< 50 years	Reference	Reference	Reference	Reference	Reference	Reference
≥ 50 years	1.82	(0.50–6.60)	1.63	(0.43–6.19)	1.64	(0.43–6.24)
Gender						
Female	Reference	Reference	Reference	Reference	Reference	Reference
Male	3.50	(1.08–11.36)	3.34	(1.02–10.96)	3.27	(0.99–10.74)
CCI group						
0	Reference	Reference	Reference	Reference	Reference	Reference
≥ 1	1.55	(0.48–5.04)	0.97	(0.29–3.30)	1.00	(0.30–3.41)
Medications						
Immunosuppressants	13.74	(3.78–49.94)	4.89	(0.94–25.33)		
Methotrexate	18.74	(4.15–84.56)				
Azathioprin	21.26	(4.71–95.91)				
Cyclophosphamide	27.86	(6.18–125.70)				
Steroid	3.94	(1.08–14.37)	2.65	(0.68–10.26)	2.72	(0.70–10.55)

*Abbreviations*: *SS* Sjögren’s Syndrome, *CCI* Charlson cormorbidity

**Table 4:** Crude and adjusted hazard ratios for the association between a variable and the risk of NTM infection within the 1st year after the diagnosis

	Univariate analysis		Model A		Model B	
	HR	(95%CI)	HR	(95%CI)	HR	(95%CI)
Group						
Non- SS	Reference	Reference	Reference	Reference	Reference	Reference
SS	16.06	(3.24–79.60)	5.22	(0.71–38.64)		
SS treated without immunosuppressants	6.28	(0.65–60.42)			4.04	(0.39–41.85)
SS treated with immunosuppressants	72.39	(12.09–433.31)			33.33	(4.37–254.23)
Age						
< 50 years	Reference	Reference	Reference	Reference	Reference	Reference
≥ 50 years	2.66	(0.31–22.76)	1.60	(0.17–15.09)	1.61	(0.17–15.25)
Gender						
Female	Reference	Reference	Reference	Reference	Reference	Reference
Male	3.81	(0.70–20.79)	2.92	(0.53–16.19)	2.85	(0.51–15.80)
CCI group						
0	Reference	Reference	Reference	Reference	Reference	Reference
≥ 1	6.89	(1.26–37.61)	4.05	(0.67–24.28)	4.16	(0.69–24.98)
Medications						
Immunosuppressants	39.16	(7.17–213.81)	6.21	(0.75–51.40)		
Steroid	6.94	(1.27–37.90)	2.55	(0.37–17.46)	2.65	(0.38–18.24)

Immunosuppressants: methotrexate, cyclophosphamide, or azathioprine. *Abbreviations*: *SS* Sjögren’s Syndrome, *CCI* Charlson comorbidity index

## Discussion

To the best of our knowledge, the present study is the first to estimate the risk of NTM infection in patients with incident SS using a nationwide, population-based dataset. The major finding of this study is that subjects with SS receiving immunosuppressive agents had a markedly increased risk for NTM infection, especially during the first year after SS diagnosis. We believe that this finding might be explained by a late diagnosis of NTM infection in a concurrent NTM infection and SS, an immunocompromised status resulting from immunosuppressant use, and the SS activity-associated vulnerability to NTM infection.

First, NTM infection may manifest with an insidious progression [6]; therefore, the occult or subclinical NTM infection may be ignored by the rheumatologist before the diagnosis of SS. Mycobacterial infection, often presenting with a subacute clinical course infection and requiring prolonged antimicrobial therapy, has been implicated to trigger autoimmunity [17–19]. Moreover, our recently published study also reported a correlation between a history of NTM infection and the risk of newly diagnosed SS, indicating that shared immunological pathways between the two conditions could explain this association [20, 21]. Thus, the high risk of NTM during the first year after SS diagnosis might at least partly be explained by the coexistence of these two diseases due to the shared immunological pathways. In Asian countries, including Taiwan, autoantibodies against interferon-gamma (IFN-γ) have been found to play an important role in patients with NTM infection [22, 23]. In addition, another recently published study, involving 150 SS patients and 199 SLE patients, found that up to 9.3% (14/150) of SS and 7.4% (14/199) of SLE patients had autoantibodies against IFN-γ [24], and more studies are warranted to determine the genetic background [25, 26].

Second, NTM are generally environmental pathogens, and the immunosuppressive agents used for treating SS may lead to an elevated risk of acquiring opportunistic NTM infections [27]. As shown in this study, methotrexate and cyclophosphamide were found to be independent risk factors for the development of NTM infection, and discussions regarding these two drugs are in the following section. Usage of immunosuppressive agents in patients with SS may increase the risk of NTM infection. However, it is truly difficult to address the risk for mycobacterial infection with one specific immunosuppressive agent in subjects with rheumatic diseases given the complexity of combinational use of traditional immunosuppressive agents and new biological agents. However, unlike the increasing use in RA and SLE, biological agents are less likely to be used in SS. In addition, current evidence of mycobacterial infection has generally relied on data from TB infection in RA or SLE [7, 28]. Brassard et al., investigating 112,300 patients with RA in one America-based database, found that traditional DMARDs were independently associated with TB (RR, 1.2; 95% CI, 1.0–1.5) [7]. However, Bogas et al. reported that methotrexate treatment in RA might not be associated with a significant increase in the incidence of TB in a Spanish population [9]. Such discrepancy may result from the distinct TB epidemiological conditions. Notably, unlike the decreasing incidence of TB infection, an increasing prevalence of NTM infection has been reported worldwide, including Taiwan [5, 29, 30]. Therefore, it is crucial to explore the impact of immunosuppressive agents on the risk of NTM infection in SS patients.

Third, usage of immunosuppressant agents in patients with SS may represent a high activity of SS, and a high activity in SS might also contribute to the development of NTM infection. Using the European League Against Rheumatism Sjögren’s Syndrome Disease Activity Index (ESSDAI) score to assess SS activity, Brito-Zeron et al. recently reported that a high SS activity was associated with infection-associated mortality in 1045 patients with SS [31]. Notably, hypocomplementemia is one key factor of the ESSDAI score for SS severity, and the activation of complement pathways has been found to be essential in the immunity against NTM [32, 33]. As shown in the present study, SS patients receiving immunosuppressive agents are vulnerable to NTM infection, and we postulate that an immunocompromised status resulting from immunosuppressive agents and a high SS activity may contribute to the development of NTM infection in these patients.

Taken these evidences together, SS was correlated with NTM infection, and early mutual surveillance between these two diseases is warranted. We believe that these data might support the need for screening for NTM infection in patients with newly diagnosed SS, particularly in those receiving immunosuppressants and presenting with unusual subacute infection-associated symptoms such as unexplained productive cough, fever, body weight loss, and poorly healing wounds.

Some limitations exist in this study that merit discussion. First, information about NTM species was lacking in this claims-based dataset; however, the data of this study merit further mechanistic studies. Second, this study excluded patients with RA or SLE; therefore, the results cannot be applied to patients with RA- or SLE-related secondary SS. Third, the accuracy of diagnoses based on claims data is of concern. However, the regular check of the quality of the claims data from all medical institutions by the BNHI has improved the coding accuracy [34] and hence minimized bias due to misclassification. Also, few patients with SS developed NTM infection, resulting in a relatively wide 95% CI; however, we thought the diagnosis of NTM infection should be accurate.

In conclusion, using a nationwide, population-based dataset, this study revealed a significant association between SS and the risk of NTM infection, in particular, in the first year after SS diagnosis. Thus, a heightened vigilance for NTM infection is required in newly diagnosed SS patients who receive immunosuppressive agents. Additional studies are warranted in the future to further investigate the underlying mechanisms.

## Additional files

Additional file 1: Table S1. Unadjusted and adjusted hazard ratios for the association between variable and the risk of NTM infection after the diagnosis. (XLSX 9945 kb)

Additional file 2: Figure S1. Kaplan-Meier survival curve for incidental NTM infection within the 1st year of SS diagnosis. (DOCX 695 kb)

Additional file 3: Dataset. Supplemental dataset. Detailed original dataset of the present study. (DOCX 25 kb)

## Abbreviations

BNHI: Bureau of national health insurance
CCI: Charlson comorbidity index
CI: Confidence intervals
ESSDAI: European league against rheumatism Sjögren’s syndrome disease activity index
HR: Hazard ratios
ICD: International classification of diseases
IFN: Interferon
IRR: Incidence rate ratios
LHID: Longitudinal health insurance database
NHI: National health insurance
NHIRD: National health insurance research data
NTM: Non-tuberculous mycobacterium
RA: Rheumatoid arthritis
RCIPD: Registry for catastrophic illness patient database
SD: Standard deviation
SLE: Systemic lupus erythematosus
SS: Sjögren’s syndrome
TB: Tuberculosis
