# Supplementary material for: The risk of nontuberculous mycobacterial infection in patients with Sjögren’s syndrome: a nationwide, population-based cohort study
Source: BMC Infect Dis. 2017 Dec 28;17:796. doi: 10.1186/s12879-017-2930-7 (PMC5745909; doi:10.1186/s12879-017-2930-7)
Supplement: Supplementary file 2 — Kaplan-Meier survival curve for incidental NTM infection within the 1st year of SS diagnosis. (DOCX 695 kb) [file 12879_2017_2930_MOESM2_ESM.docx]

**

**

**Supplementary figure 1. Kaplan-Meier survival curve for incidental NTM infection within the 1^st^ year of SS diagnosis. (**A) Categorized by SS and non-SS. (B) Categorized by non-SS, SS treated without immunosuppressants, and SS treated with immunosuppressants. NTM, nontuberculous mycobacteria; SS: Sjögren’s syndrome.
