# Supplementary material for: The risk of nontuberculous mycobacterial infection in patients with Sjögren’s syndrome: a nationwide, population-based cohort study
Source: BMC Infect Dis. 2017 Dec 28;17:796. doi: 10.1186/s12879-017-2930-7 (PMC5745909; doi:10.1186/s12879-017-2930-7)
Supplement: Supplementary file 3 — Dataset. Supplemental dataset. Detailed original dataset of the present study. (DOCX 25 kb) [file 12879_2017_2930_MOESM3_ESM.docx]

| **Supplementary table 1. Unadjusted and adjusted hazard ratios for the association between variable and the risk of NTM infection after the diagnosis** | | | | | | | | |
| --- | --- | --- | --- | --- | --- | --- | --- | --- |
|  | Univariate analysis | |  | Model A | |  | Model B | |
|  | HR | (95%CI) |  | HR | (95%CI) |  | HR | (95%CI) |
| **Group** |  |  |  |  |  |  |  |  |
| Non- SS | Reference | Reference |  | Reference | Reference |  | Reference | Reference |
| SS | 7.66 | (2.36–24.94) |  | 3.17 | (0.70–14.24) |  |  |  |
| SS treated without immunosuppressants | 2.47 | (0.31–19.53) |  |  |  |  | 1.95 | (0.24–15.68) |
| SS treated with immunosuppressants | 25.45 | (6.88–94.05) |  |  |  |  | 17.77 | (4.53–69.61) |
| **Age** |  |  |  |  |  |  |  |  |
| <50 years | Reference | Reference |  | Reference | Reference |  | Reference | Reference |
| ≥50 years | 1.82 | (0.50–6.60) |  | 1.63 | (0.43–6.19) |  | 1.64 | (0.43–6.24) |
| **Gender** |  |  |  |  |  |  |  |  |
| Female | Reference | Reference |  | Reference | Reference |  | Reference | Reference |
| Male | 3.50 | (1.08–11.36) |  | 3.34 | (1.02–10.96) |  | 3.27 | (0.99–10.74) |
| **CCI group** |  |  |  |  |  |  |  |  |
| 0 | Reference | Reference |  | Reference | Reference |  | Reference | Reference |
| ≥1 | 1.55 | (0.48–5.04) |  | 0.97 | (0.29–3.30) |  | 1.00 | (0.30–3.41) |
| **Medications** |  |  |  |  |  |  |  |  |
| Immunosuppressants | 13.74 | (3.78–49.94) |  | 4.89 | (0.94–25.33) |  |  |  |
| Steroid | 3.94 | (1.08–14.37) |  | 2.65 | (0.68–10.26) |  | 2.72 | (0.70–10.55) |
| Abbreviations: SS, Sjögren’s Syndrome; CCI, Charlson cormorbidity. | | | | | | | | |
